# Using Non-Additive Entropy to Enhance Convolutional Neural Features for Texture Recognition

**DOI:** 10.3390/e23101259

**Published:** 2021-09-27

**Authors:** Joao Florindo, Konradin Metze

**Affiliations:** 1 Institute of Mathematics, Statistics and Scientific Computing, University of Campinas, Campinas 13083-859, Brazil; 2 Faculty of Medical Sciences, State University of Campinas (UNICAMP), Campinas 13083-894, Brazil; kmetze@fcm.unicamp.br

**Keywords:** texture recognition, convolutional neural networks, non-additive entropy, image descriptors

## Abstract

Here we present a study on the use of non-additive entropy to improve the performance of convolutional neural networks for texture description. More precisely, we introduce the use of a local transform that associates each pixel with a measure of local entropy and use such alternative representation as the input to a pretrained convolutional network that performs feature extraction. We compare the performance of our approach in texture recognition over well-established benchmark databases and on a practical task of identifying Brazilian plant species based on the scanned image of the leaf surface. In both cases, our method achieved interesting performance, outperforming several methods from the state-of-the-art in texture analysis. Among the interesting results we have an accuracy of 84.4% in the classification of KTH-TIPS-2b database and 77.7% in FMD. In the identification of plant species we also achieve a promising accuracy of 88.5%. Considering the challenges posed by these tasks and results of other approaches in the literature, our method managed to demonstrate the potential of computing deep learning features over an entropy representation.

## 1. Introduction

Texture is a fundamental feature of complex digital images and texture recognition plays an important role in areas like medicine [1], material sciences [2], remote sensing [3], and many others [4].

Despite the success of learning-based approaches, especially, convolutional neural networks (CNN), texture recognition still poses some challenge, mainly when the images are collected under uncontrolled conditions. In this context, the addition of some extra information to guide the CNN algorithm has significant potential to improve the overall performance.

In parallel, an information that is known for a long time to be quite relevant in texture analysis is that provided by local patterns. Methods like that presented in the seminal study of Haralick [5] and the local binary patterns [6] are representative examples of how local-based analysis is effective, even using relatively simple strategies for that representation. More recently, in [7] we see that an interesting measure to identify local patterns is entropy. More exactly, the authors show that the non-additive Tsallis entropy is a good candidate to express several attributes of a local neighborhood, such as regularity, multifractality and complexity. All these features can collaborate for a more robust and rich statistical description of the image.

There are essentially two motivations for the use of non-additive entropy in texture analysis. The first one is that entropy is a complexity measure, which in textures is known to be related with physical properties (roughness, for example) that play important role in characterizing materials. Furthermore, non-additive entropy has connections with another successful complexity representation in texture images, which is the multifractal theory. A practical consequence of this is that such definition of entropy provides a framework for multiscale analysis without losing locality information. This substantially enriches the description of local complex patterns arising in the image and additionally providing multiscale information, which is also an important element in texture analysis models.

Based on this context, here we propose a hybrid approach that combines the local description power of non-additive entropy with the feature extraction capabilities of pretrained CNNs. More precisely, we develop two independent parallel pipelines. The first one uses the original image as input. For the second, we employ an alternative representation where each pixel is replaced by the non-additive entropy computed over a local neighborhood centered at that pixel. Both pipelines involve the subsequent application of a CNN over the input data. Such CNN is pretrained over the ImageNet and there is no fine tuning in our algorithm, in this way substantially reducing any computational overhead. Finally we take the features at the penultimate layer of the CNN at both pipelines and combine them by concatenation. These features compose our final texture descriptors.

Our approach is validated on texture classification, over classical benchmark databases (KTH-TIPS-2b [8], FMD [9], UIUC [10], and UMD [11]). An application to a specific task of plant species identification based on images of the leaf surface [12] is also accomplished. The attained results are also compared with other texture recognition methods and several state-of-the-art approaches are outperformed by the proposed descriptors. In general, such results suggest the potential of the alternative representation of texture images based on non-additive entropy, leveraging the performance of the already well-established deep learning frameworks.

## 2. Related Works

Studies like those carried out by Haralick in the 1970’s [5] and Pietikannen et al. in the 2000’s [6] consistently demonstrated the importance of local patterns for texture recognition. More specifically and more recently, the particular role of non-additive entropy as a local texture descriptor has been investigated in [7].

Inspired by the success in general tasks in computer vision in the recent years, we have also seen the rapid increase in the number of works investigating deep learning approaches to texture analysis. To mention a few examples, we have Deep Convolutional Activation Feature (DeCAF) [13], Deep Texture Encoding Network (DeepTEN) [14], Deep Filter Banks [15], Locally-Transfered Fisher Vectors (LFV) [16], Deep Texture Manifold (DEP) [17], Multiple-Attribute-Perceived (MAP) [18], and many others.

The idea of entropy has been introduced to convolutional neural networks for different purposes. For instance, in [19] the authors use information entropy for semantic-aware feature pooling. In [20], an entropy measure is employed for the quantization of different deep learning models, including CNNs. Combinations of CNNs with entropy at a high level have also been explored, for example, for malware classification [21], fault diagnosis [22], detection of epileptic seizure [23], and others.

Finally, non-additive entropies, like the Tsallis definition employed here has been used in image recognition for a long time, with examples of applications in facial recognition [24], analysis of magnetic resonance images in medicine [25], texture recognition [7,26], and so on.

## 3. Background

### 3.1. Convolutional Neural Networks

Convolutional neural networks (CNN) are artificial neural networks especially designed to process multidimensional data, such as images and videos [27]. Their most important element is the convolution operator, acting over a digital image *I* with a *kernelK* resulting in a map whose value at each position (x,y) is given by

(1) $$\mathrm{conv}{\left(I,K\right)}_{\left(x,y\right)}=\sum_{i=1}^{n_{H}}\sum_{j=1}^{n_{W}}\sum_{k=1}^{n_{C}}I\left(x+i-1,y+j-1,k\right)K\left(i,j,k\right),$$

where $n_{H}$ and $n_{W}$ are, respectively, the image height and width and $n_{C}$ is the number of convolution channels.

A typical CNN also includes other operations, such as the application of a non-linear activation function and an operation called *pooling*, which reduces the size of the map in the previous layer. A fully connected network is also frequently used on top of a set of convolutional layers, working as classifiers, whereas convolutional layers act as feature extractors. More details can be easily found in the literature [27].

The real values in the convolution kernel constitute the learnable parameters θ, which are optimized by *backpropagation*. Given a set of *m* training samples, each one corresponding to a pair input/target $\left(x_{i},y_{i}\right)$, we define an objective function by

(2) $$J\left(\theta \right)=\frac{1}{m}\sum_{i=1}^{m}L\left({\hat{y}}_{i},y_{i}\right),$$

where L is a function named *loss*, which measures the error of the network, and ${\hat{y}}_{i}$ is the output of the network, which explicitly depends on θ. Such parameters are obtained by gradient descent, an iterative numerical method where θ values are initialized at random and, in each step (*epoch*) *t*, they are updated according with

(3) $$\theta \left(t\right)=\theta \left(t-1\right)-\eta \frac{\partial J}{\partial \theta (t-1)},$$

where η is the *learning rate*.

### 3.2. Non-Additive Tsallis Entropy

Like several other definitions of the so-called generalized entropy, e.g., Boltzman, Shannon, Renyi, and others, Tsallis non-additive entropy [28] is also an adequate measure to quantify disorder or randomness of a system. More specifically, in the context of data analysis, these entropies are well known to be a powerful quantifier for the amount of information. However, Tsallis entropy was especially designed also for the purpose of identifying long-range interactions and complex dynamics.

Formally, it is defined for a probability distribution *p* by

(4) $$S_{q}\left(p\right)=\frac{k}{q-1}\left(1-\sum_{i}p_{i}\right),$$

where *q* and *k* are pre-defined parameters. In the limit q→1 we recover the classical definition of Boltzman-Gibbs-Shannon, the so-called BGS entropy:

(5) $$\lim_{q\to 1}S_{q}\left(p\right)=-k\sum_{i}p_{i}\log p_{i}.$$

## 4. Proposed Method

Despite the success of CNNs in texture representation, we still have some room for improvements, especially in the analysis of textures observed “in the wild”, i.e., under uncontrolled conditions. In this context, tasks involving texture recognition can benefit from alternative viewpoints of the same image. An example of such viewpoint that showed promising performance is the non-additive entropy of the original texture, as employed in [7]. At the same time, there is no doubt that CNNs are capable of providing powerful representation for these images, especially when coupled with some transfer learning mechanism.

Based on this, here we propose a hybrid representation for texture images that combines the local power of non-additive entropies for local description with the flexibility of a CNN acting as a feature extractor. More precisely, our method starts by computing Tsallis entropy within the neighborhood of each pixel. Formally, to each pixel at coordinates (x,y) we associate a (square) window $W_{\left(x,y\right)}^{r}$:

(6) $$W_{\left(x,y\right)}^{r}=\left\{\left(i,j\right):\left(x-r\right)\leq i\leq \left(x+r\right),\left(y-r\right)\leq i\leq \left(y+r\right)\right\}.$$

Over this region we define the histogram

(7) $$h_{\left(x,y\right)}\left(k\right)=\sum_{j=\left(k-1\right)h_{b}}^{kh_{b}}\delta \left(W_{\left(x,y\right)}^{r},j\right),$$

where $h_{b}$ is the size of the histogram bin and δ(a,b) is the Kronecker delta function: δ(a,b)=1 if a=b and 0, otherwise. The entropy is computed over such histogram. Notice that here we can disregard any constant in (4) as it would affect the entire image and would not add any descriptive element. In this sense, we simply redefine (4) as

(8) $$S_{\left(x,y\right)}^{\prime q}=\sum_{k=0}^{k_{max}}h{\left(k\right)}^{q}.$$

**Figure 1:**
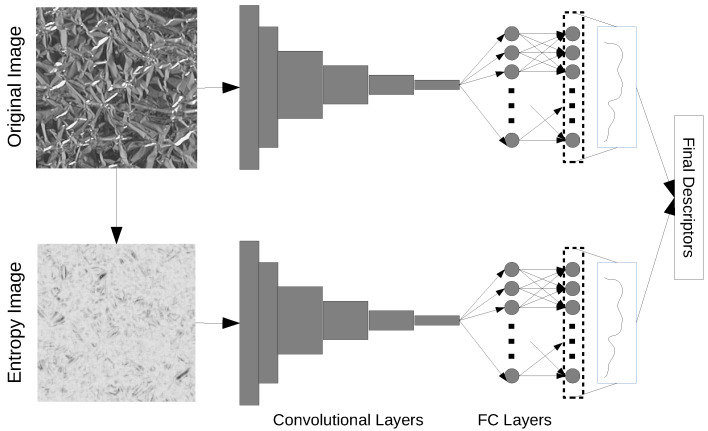
Proposed method. On top we have the pipeline for the original image, whereas at the bottom we have the processing of the image over the entropy representation. The features are collected at the penultimate layer of the CNNs and concatenated to compose the final descriptors.

Finally, a transformed image $I_{q}$ is obtained by replacing each pixel value I(x,y) by $S_{\left(x,y\right)}^{\prime q}$. $I_{q}$ is an alternative representation of *I* and may provide an interesting viewpoint over the original texture. Nevertheless, to be more effective in extracting useful features from that representation we apply $I_{q}$ as the input to a pre-trained CNN and collect descriptors at the penultimate layer (just before the *softmax* classification layer). Similar procedure can also be applied over the original image *I* and both descriptors can be combined to provide the final descriptors, which will be actually used for recognition tasks. Based on the success previously reported in texture analysis tasks [15], here we use the VGG19 (VGG-VD) architecture for the feature extractor. The diagram in Figure 1 illustrates the main steps and intermediate representations resulting from the proposed methodology.

**Figure 2:**
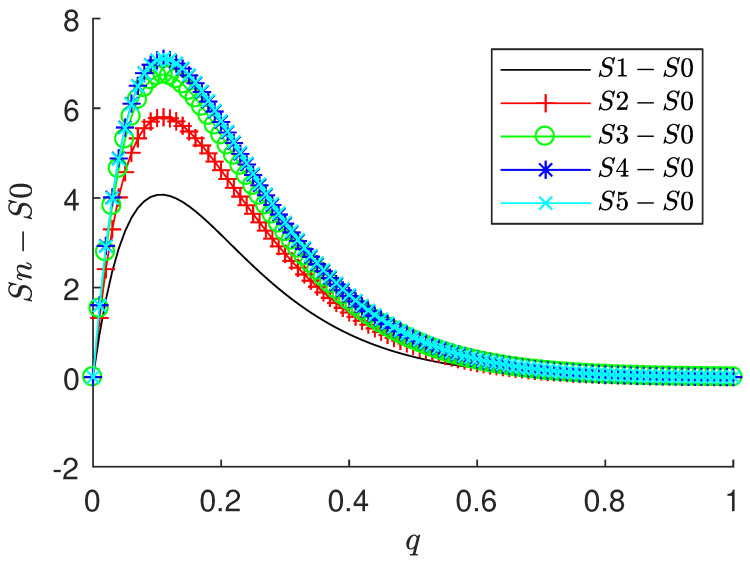
Influence of parameter *q* in changing the entropy value for perturbed distributions. The point q≈0.2 maximizes the difference between the perturbed distribution $S_{n}$ and the original one $S_{0}$.

An interesting point here is the role of the parameter *q*. In Figure 2 we have an illustration of how *q* affects entropy values when the original distribution is perturbed by some amount of noise. S0 is the entropy of the original distribution (randomly defined) and Sn is the entropy of a perturbed version of that distribution. More exactly, Sn is the entropy of the original distribution added with random values in the range [0,0.1n] and renormalized. So, a higher *n* value reflects a higher perturbation. The plot exhibits the difference Sn−S0 for n=1,2,3,4,5. It can be observed that we have an optimum value of *q* (around 0.2) that more sharply highlights the differences between pure and perturbed distribution. But smaller or larger values are expected to decrease this difference. In terms of image analysis and machine learning, such vanishing corresponds to some regularization introduced over the image descriptors. Such parameter is frequently used to control overfitting in the training process and can also be employed in the present study for that purpose. It is also important to notice that the optimum point for the *q* value is highly dependent on the distribution being processed.

The introduction of non-additive entropy to texture representation allows for a more precise analysis of local pixel patterns especially concerning multifractality. Indeed, measures like Tsallis entropy are known to be an adequate tool to describe multifractal in momentum spaces of physical systems [29]. At the same time, textures, especially those originated from natural structures (e.g., medical images), are also strongly characterized by the presence of multifractal patterns [29]. Together with the power of CNNs, we have a model capable of detecting even the most subtle patterns that otherwise could not be identified when looking only at the original image.

## 5. Validation Setup

We evaluate the performance of the proposed method over four well-established benchmark databases, to know, KTH-TIPS-2b [8], FMD [9], UIUC [10], and UMD [11].

KTH-TIPS-2b is a database of color textures with 4752 images divided into 11 balanced classes (432 samples per class). Each class is further evenly divided into 4 samples. And each sample corresponds to particular settings of acquisition, in terms of scale, pose and illumination. Each image has resolution 200×200. Here we adopt the training/testing split in [30], i.e., three samples for training and the remaining one for testing. This amounts to a total of 4 possible combinations and at the end we take the average accuracy.

FMD (Flickr Material Database) is a collection of color textures acquired under uncontrolled conditions, with a total of 1000 images equally divided into 10 classes. The resolution of the images is 512×384. The training/testing split follows the most typical protocol for texture classification, where one half of the images is randomly selected for training and the other half for testing. Such procedure is repeated 10 times and we compute the average accuracy.

UIUC (University of Illinois Urbana-Champaign) database comprises a set of 1000 gray texture images evenly divided into 25 classes. Each image has size 640×480. They are collected under uncontrolled conditions and are subject to variations in viewpoint, scale and illumination settings. The training/testing split is similar to that used in FMD.

UMD (University of Maryland) is a collection of grayscale textures which shares some similarities with UIUC, like the number of samples and classes and the acquisition conditions. The most remarkable difference is the higher resolution, which in UMD is 1280×960. We use the same training/testing split employed in UIUC and FMD.

In addition, we tested our proposal in the 1200Tex, which is a database of images corresponding to scanned photographies of the leaf surface of Brazilian plant images. The objective is to identify the respective species. The set contains 20 classes (species) with 20 samples (plant exemplars) per class. The surface image of each sample is a color texture that is partitioned into 3 non-overlapping windows with resolution 128×128. The acquisition process takes place under controlled conditions of illumination, scale and viewpoint. The training/testing split is the same one adopted for UMD, UIUC and FMD.

As for the classifier, we use Linear Discriminant Analysis (LDA) [27]. This is mainly motivated both by the fact that it does not involve any critical hyperparameter tuning and by its effectiveness in previous application to texture recognition. To reduce dimensionality and, as a consequence, the computational burden, we employ principal component analysis before the input to the classifier. The number of principal components is determined by 5-fold cross-validation over the training set. We set a maximum possible of 200 components.

## 6. Results and Discussion

In this section, we present results on the application of the proposed method to texture classification over the benchmark databases and on the application to plant species identification.

**Figure 3:**
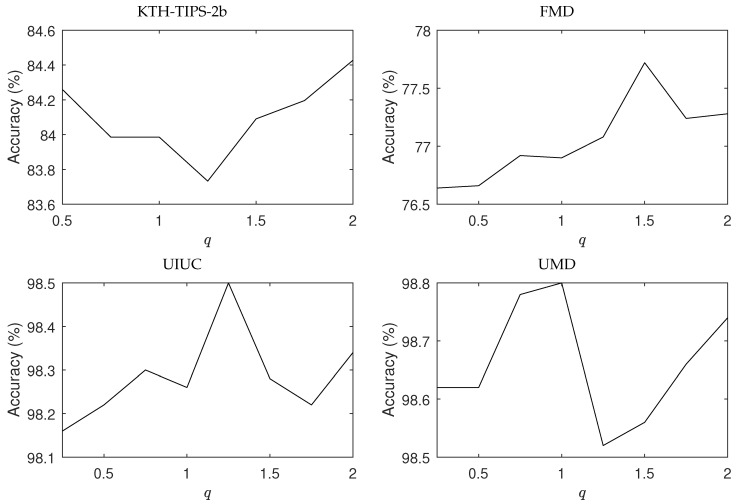
Accuracy on the benchmark databases for different values of *q*. Except for KTH-TIPS-2b, all the other databases present compatible behavior, with the best performance achieved at intermediate values of *q*.

The accuracies in the benchmark datasets for different values of *q* are presented in Figure 3. The original color image was used for the color textures (KTH-TIPS-2b, FMD, and 1200Tex) in the pure CNN pipeline and the grayscale version in the entropy input. In practice, and to provide reliability to the choice of the optimum value of *q*, a validation set separated from the training images can be employed, following the usual protocol in machine learning. One possibility for that is to employ a K-fold split, where the training images are randomly divided into *K* subsets (e.g., K=5), each one with roughly the same number of images. We selected one subset for validation and the remainder K−1 subsets for training. At the end we computed the average accuracy for different values of *q* and took that value yielding the best performance. Figure 3 is an example of such analysis. The highest accuracy attained by the proposed descriptors are, respectively, 84.5% for KTH-TIPS-2b with q=2.0, 77.7% for FMD with q=1.5, 98.5% for UIUC with q=1.25, and 98.8% for UMD with q=1.0. We observe two basic distinct behaviors: while extreme values of *q* (around 0.5 and 2.0) was better suited for KTH-TIPS-2b, an intermediate value around 1.5 was the preferred choice for the other benchmark databases. As well illustrated in Figure 2, *q* essentially controls the regularization of the original descriptors. In this context, the results for KTH-TIPS-2b attests that those textures are highly affected by regularization. This is an expected consequence considering the high inter-sample variability, which requires the classifier to identify subtle patterns. Regularization is not effective in this scenario.

**Figure 4:**
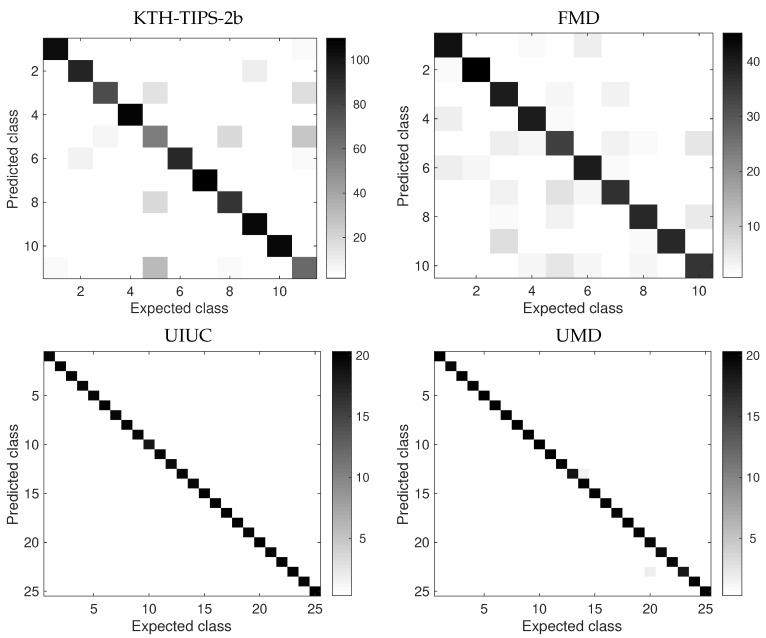
Confusion matrices on the benchmark databases. As expected from results in Figure 3, UIUC and UMD present no significant error, while the errors in KTH-TIPS-2b and FMD correspond to samples from similar materials or with high intra-class variability.

The confusion matrices of the four benchmark databases analyzed are shown in Figure 4. Looking at the performance of the proposed descriptors on different classes allows for interesting observations. First, as supposed from the high accuracy in Figure 3, there is no relevant confusion on UIUC and UMD. On the other hand, the scenario is substantially more challenging in KTH-TIPS-2b and FMD. In the first one we have visible problems with classes 5, 8 and 11. Those correspond, respectively, to images of the materials cotton, linen and wool. These are different types of fabrics and certainly share several common pixel patterns, which make even the visual distinction challenging. FMD, on its turn, presents more homogeneous distribution of errors, even though we still notice some prevalence on classes 3, 5 and 10 (glass, metal and wood). In this case the probable cause is the high variability of color and shape present in those samples.

Table 1 lists the accuracy of a collection of methods for texture analysis in the literature compared with the proposed approach. In general, our descriptors were capable of outperforming several state-of-the-art approaches in all the compared databases. This is a particularly interesting achievement if we consider that our strategy is relatively simple. It also confirms the importance of complexity to represent textures even in the deep learning framework. Actually, even though the role of such features is well studied in classical texture analysis, modern learning-based approaches usually rely on the idea that all useful information should be automatically discovered by the CNN. Our studies demonstrate that this is not always the best solution and the alternative representation provided by measures like entropies can still be useful for a more holistic representation.

**Figure 5:**
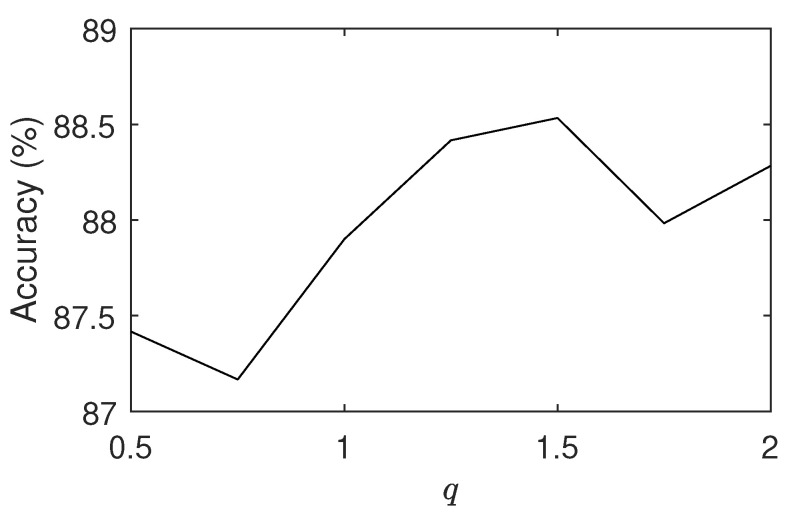
Accuracy on the 1200Tex database for different values of *q*. Similar to what was observed in Figure 3, here we also have the highest accuracy for an intermediate value of *q*.

The results for the application to the problem of plant species identification are presented in separated figures and tables, given that most methods compared in the benchmark databases do not have results published for the 1200Tex dataset. Figure 5 shows the effect of using different values of *q* on the plant images problem. The behavior is similar to most benchmark cases in Figure 3 and we had an optimum value q=1.5, yielding an accuracy of 88.5%. Indeed, 1200Tex comprises relatively homogeneous textures and regularization plays an important role in preventing that spurious details contaminate the overall performance.

**Figure 6:**
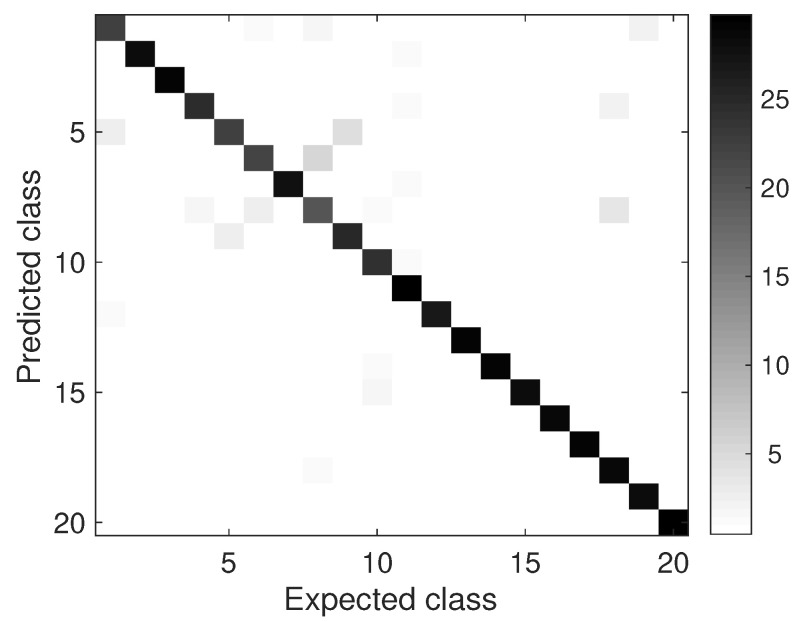
Confusion matrix on the 1200Tex database. The most relevant errors concentrate around classes 6–10 and 18, which correspond to similar nervure patterns, a widely accepted discriminative trait in botany.

Figure 6 depicts the confusion matrix for the plant problem. Here we notice some significant errors in classes 6–10 and 18. Those samples are characterized by quite similar patterns of leaf nervures. This is known to be an important trait in botany [46] and the confusion in this scenario was in some sense expected.

Table 2 compares the accuracy of the proposed method in the 1200Tex database with other ones in the literature. Again, our approach managed to achieve promising performance, even when compared with some computationally intensive approaches like the Fisher vectors over CNN (FV-CNN) developed by Cimpoi et al. [15]. The results also confirm that our expectation suggested by the benchmark results in Table 1 are also true in a real-world task of practical importance.

The computational time does not depend on parameter *q*. If several values of *q* are tested, this will linearly increase the time, but such tests are not carried out over the entire database, but rather over only the validation set. As for the image size, the computational time depends on the number of pixels. For the square images with dimension n×n processed here, the order of complexity is $O(n^{2})$. We should also observe that such time is not relevant if put in perspective with the deep learning subsequent processing.

In summary, the results presented in this section suggest that the combination of the original image with an alternative representation in the space of local non-additive entropies has potential to significantly improve the accuracy in texture analysis. The evaluation on datasets with quite different characteristics also suggests the flexibility and robustness of the proposed descriptors with respect to the most diverse variations in attributes like illumination, viewpoint, scale, and others, present in the analyzed databases.

## 7. Conclusions

This study proposed and investigated the performance of a texture descriptor combining features extracted by a pre-trained convolutional neural network over the original image with features extracted in similar way but over an alternative representation where each pixel is replaced by a measure of local non-additive entropy.

The potential of our approach was verified in texture classification, both on benchmark databases and on a practical task of identifying plant species based on the scanned image of plant leaf surfaces. In all situations, our method demonstrated its potential as a robust and precise descriptor, outperforming several approaches of the state-of-the-art in the area.

We can summarize saying that our findings confirm that complexity measures, like the non-additive entropy investigated here, have the potential to take advantage of the modern learning-based approaches in texture analysis, especially in the most challenging scenarios, where the extra information captured in the transformed space is highly effective in identifying and characterizing even the most subtle visual patterns.

## Figures and Tables

**Table 1:** Accuracies for different databases: KTH-TIPS-2b, FMD, UIUC, and UMD according to several published works. Our proposed method outperforms a number of modern approaches in texture recognition, including learning-based models.

Method	KTH2b	FMD	UIUC	UMD
VZ-MR8 [31]	46.3	22.1	92.9	-
VZ-Joint [32]	53.3	23.8	78.4	-
BSIF [33]	54.3	-	73.4	96.1
CLBP [34]	57.3	43.6	95.7	98.6
ScatNet (NNC) [35]	63.7	-	88.6	93.4
DeCAF [36]	70.7	60.7	94.2	96.4
SIFT + BoVW [36]	58.4	49.5	96.1	98.1
FC-CNN VGGM [15]	71.0	70.3	94.5	97.2
FC-CNN AlexNet [15]	71.5	64.8	91.1	95.9
FC-CNN VGGVD [15]	75.4	77.4	97.0	97.7
RAMBP [37]	68.9	46.8	94.8	98.6
H2OEP [38]	64.2	-	-	-
SWOBP [39]	66.4	-	-	-
SLGP [40]	53.6	-	-	-
LBPC [41]	50.7	-	-	-
LETRIST [42]	65.3	-	97.7	98.8
BRINT${}_{CPS}$ [43]	-	-	92.2	93.5
MRELBP${}_{CPS}$ [43]	-	-	95.2	94.2
DSTNet [44]	61.0	-	93.6	98.5
2D-LTP [45]	-	49.0	-	-
Proposed	84.4	77.7	98.5	98.8

**Table 2:** State-of-the-art accuracies for 1200Tex. The proposed method outperforms even some complex and computationally intensive algorithms like the deep learning FV-CNN approach.

Method	Accuracy (%)
LBPV [47]	70.8
Network diffusion [48]	75.8
FC-CNN VGGM [15]	78.0
FV-CNN VGGM [15]	83.1
Gabor [12]	84.0
FC-CNN VGGVD [15]	84.2
SIFT + BoVW [36]	86.0
FV-CNN VGGVD [15]	87.1
DSTNet [44]	79.3
CATex [49]	84.7
VisGraphNet [50]	87.3
Proposed	88.5

## Data Availability

Not applicable.
